# Prediction of Air Pollutants Concentration Based on an Extreme Learning Machine: The Case of Hong Kong

**DOI:** 10.3390/ijerph14020114

**Published:** 2017-01-24

**Authors:** Jiangshe Zhang, Weifu Ding

**Affiliations:** 1 School of Mathematics and Statistics, Xi’an Jiaotong University, Xi’an 710049, China; jszhang@mail.xjtu.edu.cn; 2 School of Mathematics and Information, BeiFang University of Nationalities, Yinchuan 750021, China

**Keywords:** feed forward neural network, air pollution, back propagation, extreme learning machine, prediction

## Abstract

With the development of the economy and society all over the world, most metropolitan cities are experiencing elevated concentrations of ground-level air pollutants. It is urgent to predict and evaluate the concentration of air pollutants for some local environmental or health agencies. Feed-forward artificial neural networks have been widely used in the prediction of air pollutants concentration. However, there are some drawbacks, such as the low convergence rate and the local minimum. The extreme learning machine for single hidden layer feed-forward neural networks tends to provide good generalization performance at an extremely fast learning speed. The major sources of air pollutants in Hong Kong are mobile, stationary, and from trans-boundary sources. We propose predicting the concentration of air pollutants by the use of trained extreme learning machines based on the data obtained from eight air quality parameters in two monitoring stations, including Sham Shui Po and Tap Mun in Hong Kong for six years. The experimental results show that our proposed algorithm performs better on the Hong Kong data both quantitatively and qualitatively. Particularly, our algorithm shows better predictive ability, with $R^{2}$ increased and root mean square error values decreased respectively.

## 1. Introduction

Currently, the environmental problem may be the most severe problem which has a great influence on human health and ecosystems. The governments have put great efforts towards the control of pollution, and have obtained much success. Because of the use of gasoline and other petrochemicals and fossil fuels, air pollutants are emitted largely by industry and automobiles. The formation of air pollutants is a very complex and nonlinear phenomenon, due to photochemical processes.

Air pollution degrades air quality and leads to several diseases, such as asthma, wheezing, and bronchitis. Air Pollutant is formed in the atmosphere because other directly emitted pollutants react. While Air Quality System monitoring data are viewed as the gold standard for characterizing ambient air quality and determining compliance with the government Ambient Air Quality Standards, such data are limited in space and time. The prediction of the concentration of air pollutants can enhance the scientific understanding of air pollution and provide valuable information for the development of optimal emission control strategies [1,2,3,4,5]. This predictive ability would also provide a better understanding of the nature and relative contributions of different emission sources that are responsible for the observed level of air pollutants. The system which is able to predict the concentration of air pollutants with sufficient anticipation can provide public authorities the time required to manage the emergency. Great progress has been made in the prediction of the concentration of air pollutants over the past decades. However, it is still challenging to accurately predict the concentration of air pollutants due to the complex influential factors. It is necessary to study more effective methods to accurately predict the concentration of air pollutants in the future.

The methods for the prediction of the concentration of air pollutants can be roughly divided into two types: deterministic and stochastic. The deterministic approaches model the physical and chemical transportation process of the air pollutants in terms of the influences of meteorological variables, such as wind speed, relative humidity, and temperatures with mathematical models to predict the level of air pollutants [6]. These methods can generate either short-term or long-term pollutant concentration predictions. The performance of these models depends on a thorough understanding of the formation mechanism of pollutants. Some researchers try to develop and improve an integrated air quality modeling system that can simulate the sources, evolution, and environmental impacts of air pollutants at all scales. However, it is still challenging to precisely predict the concentration of air pollutants, due to the multiplicity of sources and the complexity of the physical and chemical processes which affect the formation and transportation of air pollutants. Firstly, the parameters in the equations have a vital influence on the prediction performance. Consequently, the complexity of the large partial differential equations is high—they are very difficult to solve exactly and will sacrifice great computation resources. Meanwhile, the density and quality of observations which are used as inputs to the model also affect the accuracy of numerical predictions.

A statistical approach learns from historical data and predicts the future behaviour of the air pollutants. Many statistical models are adopted to predict the concentration of air pollutants in space and time as related to the dependent variables [7,8,9,10,11]. Some researchers proposed an exploitation of the statistical relationships between the concentration of air pollutants and the corresponding meteorological variables. It is not necessary to model a physical relationship between emissions and ambient concentrations, but to analyze the time series directly. The representative methods include time series analysis, Bayesian filter, artificial neural networks, etc. Although statistical models can present accurate prediction, they cannot provide a detailed explanation of the air pollution [12,13,14,15]. The spatial temporal interpolation is the most popular algorithm in the predictions, and is based on the assumption that the nearer two points are, the higher correlation they are [16]. It firstly analyzes the correlation of the sampled data and then uses the correlation to predict the concentration in the future [17]. However, these methods do not consider the transformation of the air pollutants in two adjacent times. Thus, the dynamical information are not considered. Some researchers proposed the combination of the observation and the output of the numerical weather system and obtain the fused estimation of the concentration of air pollutants in the Bayesian framework [18]. However, the unanalytic formation of the posterior distribution is generally solved by Markov Chain Monte Carlo (MCMC) methods in which the parameters are generally difficult to determine.

Meteorological conditions significantly affect the levels of air pollution in the urban atmosphere, due to their important role in the transport and dilution of pollutants. It has also been concluded that there is a close relationship between the concentration of air pollutants and meteorological variables. Thus, multiple linear regression models (MLR) are trained based on existing measurements and are used to predict future concentrations of air pollutants in the future according to the corresponding meteorological variables. Well-specified regressions can provide reasonable results. However, the reactions between air pollutants and the influential factors are highly nonlinear, leading to a highly complex system of air pollutant formation mechanisms. Therefore, although multiple linear regressions are theoretically sophisticated for forecasting, they are not widely used in many applications. Moreover, the outliers and the noise in the data have a strongly negative influence on the performance of these regression-based algorithms. Statistical techniques do not consider individual physical and chemical processes, and use historical data to predict the concentration of air pollutants in the future. It is very challenging to predict air quality using a simple mathematical formula which is unable to capture the non-linear relationship among various variables.

Black box approaches have been recognized as perfect alternatives to traditional models for input–output mathematical models. It is shown that neural networks show better performances against MLR [19,20,21,22,23,24,25]. Artificial neural networks (ANN) have the advantages of incorporating complex nonlinear relationships between the concentration of air pollutants and the corresponding meteorological variables, and are widely used for the prediction of air pollutants concentration. However, ANN-based approaches have the following main drawbacks: (1) ANN-based approaches very easily fall into the trap of local minimum and have poor generalization; (2) they lack an analytical model selection approach; (3) it is very time-consuming to find the best architecture and its weights by trial and error.

According to the above superiority, we proposed the use of an extreme learning machine (ELM) [26,27,28] to efficiently predict the concentration of air pollutants. To the best of our knowledge, there are no declarations that use ELM to predict the concentration of air pollutants. Our paper has two main contributions: (1) the prediction of the concentration of air pollutants in the framework of ELM. It is concluded that ELM has stronger generalization than traditional statistical and ANN-based methods, with extreme learning speed. In the second part, a brief introduction of the ELM is given and we propose the prediction of the concentration of air pollutants based on ELM simultaneously [29]; (2) ELM is evaluated on the Hong Kong data qualitatively and quantitatively in the third section comparing ELM with a feedforward neural network based on back propagation (FFANN-BP) and MLR. In the last section, we conclude our work and make some comments on future work.

## 2. Study Area

Hong Kong is located on China’s south coast, with around 7.2 million inhabitants of various nationalities, and is surrounded by the South China Sea on the east, south, and west, and borders the Guangdong city of Shenzhen to the north over the Shenzhen River. It has a land area of 1104 km^2^, is one of the world’s most densely populated metropolises, and consists of Hong Kong Island, the Kowloon Peninsula, the New Territories, and over 200 offshore islands, of which the largest is Lantau Island. In Hong Kong, millions of people live and work near heavily travelled roads. Summer is hot and humid with occasional showers and thunderstorms, and with warm air coming from the southwest, typhoons most often occur. The occasional cold front brings strong, cooling winds from the north. It is generally sunny and dry in Autumn. The most temperate seasons are spring, which can be changeable. The highest and lowest ever recorded temperatures across all of Hong Kong, on the other hand, are 37.9 °C at Happy Valley on 8 August 2015 and −6.0 °C at Tai Mo Shan on 24 January 2016, respectively. The primary pollutants are carbon monoxide and sulfur dioxide emissions from vehicles and power plants.

In a rapidly changing city like Hong Kong, traffic volume, regulations, and related policies have a great influence on the formation of air pollutants. Marine vessels and power plants are the influential factors of Hong Kong’s air pollution. The emissions of power stations, and domestic and commercial furnaces all contribute to the air pollution in Hong Kong. Smog is caused by a combination of pollutants—mainly from motor vehicles, industry, and power plants in Hong Kong and the Pearl River Delta. Approximately 80 % of the city’s smog originates from other parts of the Pearl River Delta. Air quality has deteriorated seriously in Hong Kong as a result of urbanization and modernization. Because of the reduction of air quality, cases of asthma and bronchial infections have recently increased. The mortality rate from vehicular pollution can be twice as high as near heavily travelled roads. Thus, city residents face a major health risk. Meanwhile, the pollution is costing Hong Kong financial resources. The Environment Bureau of Hong Kong has been implementing a wide range of measures locally to reduce the air pollution. The objective of overall policy for air quality management in Hong Kong is to achieve as soon as reasonably practicable and to maintain thereafter an acceptable level of air quality to safeguard the health and well being of the community, and to promote the conservation and best use of air in the public interest.

**Figure 1:**
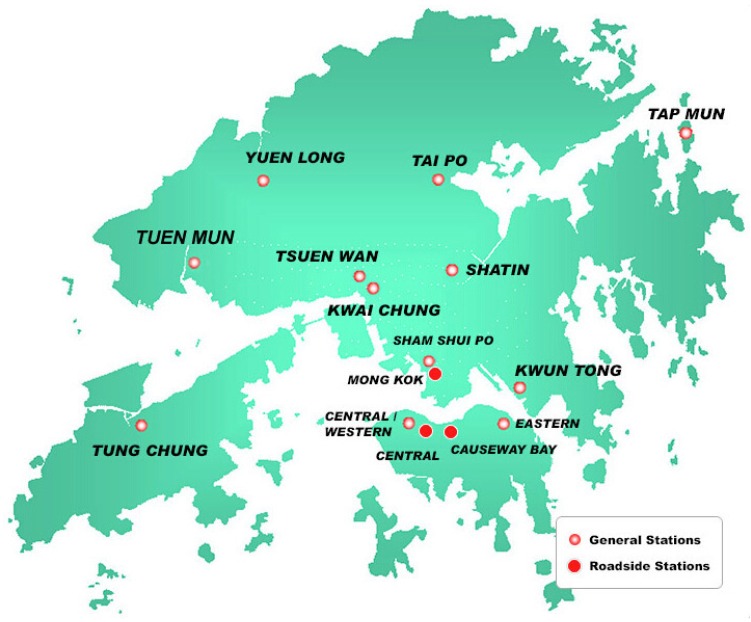
The locations of air monitoring stations in Hong Kong.

Air quality monitoring by the Environmental Protection Department is carried out by 12 general stations and three roadside stations, including Causeway Bay, Central, Central Western, Eastern, Mong Kok, Tung Chung, Shatin, Sham Shui Po, Kwai Chung, Kwun Tong, Tai Po, Tap Mun, Tsuen Wan, and Yuen Long air monitoring stations. The coordinates of monitoring stations are shown in Figure 1. The department began reporting data on fine suspended particulate—which are a leading component of smog in the air—on an hourly basis. The seasons are defined as summer (March, April, and May), monsoon (June, July, August), post-monsoon (September, October, and November), and winter (December, January, and February). The descriptive statistics of air pollution in four season are shown in Table 1, the winter and summer have the highest percentage.

## 3. Prediction of the Concentration of Air Pollutants Based on ELM

Meteorological conditions have a large and significant influence on the level of air pollutant concentrations in the urban atmosphere due to their important role in the transport and dilution of the pollutants. ELMs have become a hot area of research over the past years and have been proposed for both generalized single-hidden-layer feedforward and multi-hidden-layer feedforward networks. It has been becoming a significant research topic for artificial intelligence and machine learning because of fast training and good generalization. It seems that ELM performs better than other conventional learning algorithms in applications with higher noise.

### 3.1. Multiple Linear Regression

Multiple linear regression (MLR) tries to model the explanatory variables and response variables through fitting a linear relationship to observed data. That is to say,

(1) $$y_{t}=\beta_{0}+\beta_{1}x_{1t}+\ldots +\beta_{p}x_{pt}+\varepsilon_{t}$$

$\varepsilon_{t}$ represents the residual term, which is normally distributed with mean 0 and variance *σ*. The coefficients $\beta =(\beta_{0},\beta_{1},\ldots ,\beta_{p})$ are calculated by minimizing the sum of the squares error from each data point to the optimal value.

### 3.2. Feedforward Neural Network Based on Back Propagation (FFANN-BP)

Inspired by biological neural networks, artificial neural networks are used to approximate functions that depend on a large number of inputs. The basic structure of artificial neural networks includes a system of layered, interconnected nodes. Feed forward artificial neural networks are a simplified mathematical model based on the knowledge of the human brain neural network from the perspective of information processing, and have been found to perform remarkably well in capturing complex interactions within the given input parameters with satisfactory performance.

FFANN-BP is the most popular and the widely-used supervised learning method, and requires a teacher who knows the desired output for any given input. FFANN-BPs are systems of interconnected neurons that exchange messages between each other, in which the connections have numeric weights that can be tuned based on experience. They consist of an input layer and a hidden layer, the output layer. Thus making FFANN-BPs adaptive to inputs and capable of learning. The learning process repeats until the error of neural network decreases to the desired minimum.

The factors that influence the pollution concentration are classified and detected cautiously and then used as the input data, and the concentrations are used as the output to train the neural networks. FFANN-BPs can accurately represent the relationships between the influential factors and the air pollution concentration which are not fully captured by the traditional approaches, and can be used to predict the air pollutant concentration with the known influential factors.

The training process of FFANN-BPs consists of two iterative steps, including the forward-propagating of the data stream and the back-propagating of the error signal. Firstly, original data are passed from the input layer to the output layer through the hidden processing layer. The input of the *j*-th neuron in the *l*-th layer $x_{j}^{l}$ is

(2) $$x_{jq}^{l}=\sum_{i}w_{jiq}^{l}y_{iq}^{l-1}$$

where $w_{jiq}^{l}$ is the weight that connects the *i*-th neuron in the l−1-th layer and the *j*-th neuron in the *l* layer, $y_{jq}^{l}=f\left(x_{jq}^{l}\right)-\theta_{jq}^{l}$ is the response of the *j*-th neuron in the *l*-th layer, and *f* is the activation function which is used to introduce the non-linearity into the network. Generally, any nonlinear function can be used as the activation function, such as the unit step function; the linear function and the sigmoid function, $\theta_{jq}^{l}$, is the bias of the neuron. If the real output is not consistent with the desired output, then error is propagated backward through the network against the direction of forward computing. The learning process consists of forward and backward propagations. FFANN-BP dynamically searches the weight which minimizes the network error in the weight space, reaches the aim of the memory process and the information extraction, and makes the real output of the network closer to the desired output.

According to the convenience of the calculation, $\theta_{jq}^{l}$ can be considered as the weight of the neuron whose response is constant with −1.

The total error of the network is

(3) $$E=\frac{1}{2}\sum_{q=1}^{m}\sum_{j=1}^{n_{L}}{\left(y_{jq}^{L}-o_{jq}\right)}^{2}$$

where $o_{jq}$ is the target output of the *j*-th neuron in the output layer for the *q*-th sample, $y_{jq}^{L}$ is the real output, *m* is the number of training samples, and *L* is the number of layers for neural network. The biases can be adjusted according to the adjusting rules of the neurons. The connected weights in the output layer can be updated online according to the following formula:

(4) $$w_{jiq}^{L}\left(t+1\right)=w_{jiq}^{L}\left(t\right)+\varepsilon \left(-\nabla_{w_{jiq}^{L}}E\right)=w_{jiq}^{L}\left(t\right)+\epsilon \frac{dy_{jq}^{L}}{dx_{jq}^{L}}\left(y_{jq}^{L}-o_{jq}\right)y_{iq}^{L-1}=w_{jiq}^{L}\left(t\right)+\epsilon \delta_{j}^{L}y_{iq}^{L-1}$$

where $\delta_{j}^{L}$ is defined as

(5) $$\delta_{j}^{L}=\frac{dy_{jq}^{L}}{dx_{jq}^{L}}\left(y_{jq}^{L}-o_{jq}\right)$$

The connected weights in the hidden layers are updated with the following formula,

(6) $$w_{jiq}^{l}\left(t+1\right)=w_{jiq}^{l}\left(t\right)+\epsilon \delta_{j}^{l}y_{iq}^{l-1}$$

(7) $$\delta_{jq}^{l}=\frac{dy_{jq}^{l}}{dx_{jq}^{l}}\sum_{s=1}^{n_{l}}\delta_{s}^{l+1}w_{sjq}^{l+1}$$

where $n_{l}$ is the number of the neurons in the *t*-th hidden layer.

Three key drawbacks of FFANN-BP may be: (1) slow gradient-based learning algorithms are extensively used to train neural networks. It is clear that the learning speed of feedforward neural networks is in general far slower than required, and it has been a major bottleneck in their applications for past decades. The overtraining of the neural network results from FFANN-BP. Good performance is time-consuming in most applications due to the gradient-based optimization; (2) All the parameters of the networks are tuned iteratively by using such learning algorithms. When the learning rate *η* is too small, the convergence of FFANN-BP is too slow. When the learning rate *η* is too large, the performance of the algorithm is not stable, even divergence; (3) FFANN-BP is always prone to get caught up in a local minimum, not satisfying the performance requirements.

### 3.3. Prediction of the Concentration of Air Pollutants Based on ELM

**Figure 2:**
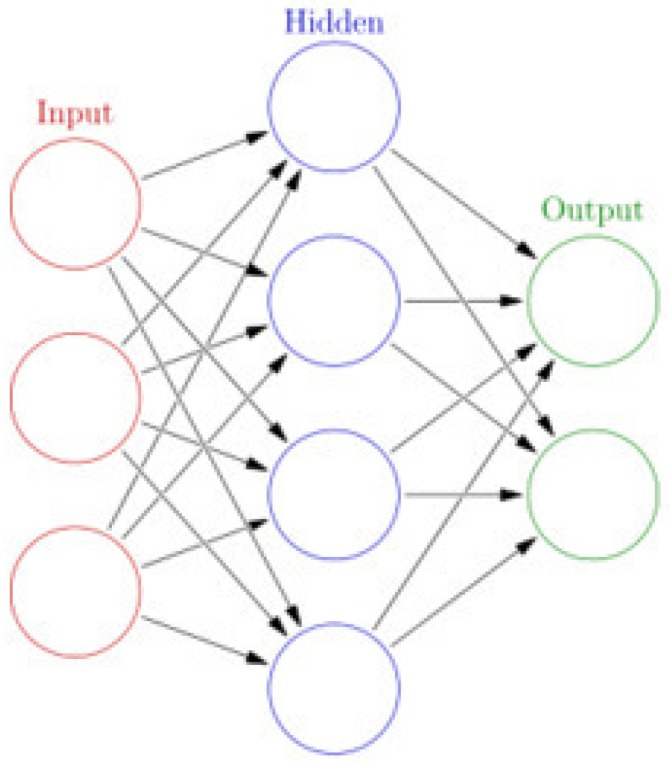
The structure of extreme learning machine (ELM). The parameters of the hidden layer are randomly generated, and the parameters of the output layer are adjusted by least squares algorithm.

ELM is basically a two-layer neural network in which the first layer is fixed and random, and the second layer is trained. The basic structure of ELM is shown in Figure 2. ELM has recently been used for classification and regression, clustering, feature selection, etc. Hardware implementation and parallel computation techniques guarantee the training of ELM. ELM has been widely used in a variety of areas, such as biomedical engineering and computer vision. Many researchers from every corner of the world pay great attention to finding an effective learning algorithm to train neural networks by adjusting hidden layers. ELM shows that hidden neurons are important but need not be tuned in many applications, which is proposed based on our intuitive belief in biological learning and neural networks’ generalization performance theories, in which the weights connecting inputs to hidden nodes are randomly assigned and never updated because of the randomly generated hidden nodes.

It is different with other machine learning algorithms, such as supported vector machine (SVM) [30] and deep learning [31]. SVM uses a kernel function to implement the feature mapping. In deep learning, one uses Restricted Boltzmann machines or Auto-Encoders/Auto-Decoders for feature mapping. It is different with traditional learning algorithms such as FFANN-BP, in which the parameters of hidden layers and the output layer all need to be adjusted. In ELM, the weights of hidden layers need not to be adjusted.

The training of ELM generally consists of two main stages, including random feature mapping and linear parameters solving. In the second stage, the output weight **fi** is calculated.

Given *N* different samples $\left(x_{i},t_{i}\right)$, where $x_{i}={\left[x_{i1},x_{i2},\ldots ,x_{in}\right]}^{T}\in R^{n}$ and $t_{i}={\left[t_{i1},t_{i2},\ldots ,t_{im}\right]}^{T}\in R^{m},i=1,\ldots ,N$. In our study, $t_{i}$ is the *i*-th air pollutants concentration, and $x_{i}$ is the corresponding meteorological variables. The neural network has $\tilde{N}$ hidden nodes, $\tilde{N}\leq N$. One first randomly assigns input weight $w_{i}$ and bias $b_{i}$, and hidden node number $\tilde{N}$, maps the input data nonlinearly into a feature space by the specified transform activation function g(x), and obtains the hidden layer output matrix H. The weight vector $w_{i}={\left[w_{i1},w_{i2},\ldots ,w_{in}\right]}^{T}$ connects the *i*-th hidden neuron and the input neurons, and the weight vector $\beta_{i}={\left[\beta_{i1},\beta_{i2},\ldots ,\beta_{im}\right]}^{T}$ connects the *i*-th hidden neuron and the output neurons; $b_{i}$ is the threshold of the *i*th hidden neurons. Compared with FFANN-BP, the input weights and the biases of the hidden layer are first randomly generated, and then the output weights are analytically adjusted through simple generalized inverse operation of the hidden layer output matrices in ELM. This is equivalent to minimizing the cost function

(8) $$E=\sum_{j=1}^{N}{\left(\sum_{i=1}^{\tilde{N}}\beta_{i}g\left(w_{i}x_{i}+b_{i}\right)-t_{j}\right)}^{2}$$

It is undesirable that the learning algorithm stops at a local minima if it is located far above a global minima. Thus, the weights between the hidden layer and the output layer are the only parameters needing to be tuned. It has been proven that the standard single layer forward networks with $\tilde{N}$ hidden nodes and activation function g(x) can approximate these *N* samples with error and give sufficient training error for any given training set with probability one. That is to say, there theoretically exist the weight vector $\beta_{i},w_{i}$ and threshold $b_{i}$ such that

(9) $$\sum_{i=1}^{\tilde{N}}\beta_{i}g\left(w_{i}x_{j}+b_{i}\right))=t_{j},j=1,2,\ldots ,N.$$

We write the above *N* equations compactly as follows:

(10) Hβ=T

where

(11) $$H\left(w_{1},\ldots ,w_{\tilde{N}},b_{1},\ldots ,b_{\tilde{N}},x_{1},\ldots ,x_{N}\right)={\left[\begin{array}{ccc} g(w_{1}\cdot x_{1}+b_{1}) & \ldots & g(w_{\tilde{N}}\cdot x_{1}+b_{\tilde{N}})\\ \vdots & \ldots & \vdots \\ g(w_{1}\cdot x_{N}+b_{1}) & \ldots & g(w_{\tilde{N}}\cdot x_{N}+b_{\tilde{N}}) \end{array}\right]}_{N\times \tilde{N}}$$

Theoretically, any output functions may be used in different hidden neurons. However, it is necessary to satisfy the universal approximation capability theorem.

In the second stage of ELM training, we found the weights connecting the hidden layer and the output layer

(12) $$\beta^{*}=H^{†}T$$

where $H^{†}$ represents the Moore–Penrose generalized inverse of matrix H,

(13) $$H=\left[\begin{array}{c} h(x_{1})\\ \vdots \\ h(x_{N}) \end{array}\right]=\left[\begin{array}{ccc} h_{1}\left(x_{1}\right) & \cdots & h_{L}\left(x_{1}\right)\\ \vdots & \vdots & \vdots \\ h_{1}\left(x_{N}\right) & \cdots & h_{L}\left(x_{N}\right) \end{array}\right]$$

is the hidden layer output randomized matrix, and T is the training data target matrix,

(14) $$T=\left[\begin{array}{c} t_{1}^{T}\\ \vdots \\ t_{N}^{T} \end{array}\right]=\left[\begin{array}{ccc} t_{11} & \cdots & t_{1m}\\ \vdots & \vdots & \vdots \\ t_{N1} & \cdots & t_{Nm} \end{array}\right]$$

The learning stability is also considered in ELM. ELMs have more generalization ability, and aim to reach the global maximum solution. ELMs not only achieve state-of-the-art performances, but also speed up the training of the network. It is difficult to achieve such performance by conventional learning techniques.

It is noted that there are no biases in the output nodes which will result in suboptimal solutions. Moreover, the number of the hidden neurons is smaller than the number of distinct training samples. The activation function of the hidden neurons is generally continuous and differentiable in the traditional feed forward neural network. FFANN-BP is quite essentially different from MLR. However, each of them can be adjusted to suit the specific applications.

## 4. Experiments

The performance of MLR, FFANN-BP, and ELM are evaluated on Hong Kong data sets which are observed from Hong Kong Observatory (HKO) and Environmental Protection Department (EPD). Because of the performance of the instruments, the data sets are not noise -free. The effectiveness of the data are poor, and the incompleteness of data has a limitation on our study. In this study, six year daily data (2010–2015) of five air pollutants at Sham Shui Po and Tap Mun air quality monitoring stations in Hong Kong was used to evaluate the accuracy of the above-mentioned statistical techniques. The air quality variables used in this study are nitrogen dioxide (NO_2_), nitrogen oxide (NO_*x*_), ozone (O_3_), particulate matter under 2.5 μm (PM_2.5_), and sulfur dioxide (SO_2_). We took the average of 24 h concentration as the daily mean concentration. All the values are in μg/m^3^. We deleted all NAs (missing values) in the data set. Eleven predictor variables and one response variable were used, which is the next day’s air pollutant concentration. For each pollutant, NAs and outliers are about 3%.

Similarly, meteorological parameters were recorded on a daily basis. Hence, the 24 hourly averaged surface meteorological variables such as daily maximum temperature, minimum temperature, difference between daily maximum and minimum temperature, average temperature (T in °C), wind speed (WS in m/s), wind direction (WD in rad), relative humidity, and three time variables such as day of the week and month of the year as inputs for three machine learning models, observed in Sham Shui Po and Tap Mun and acquired from Hong Kong observatory for the period from 2010 to 2015. The influential factors are selected by the a priori knowledge of the characteristics of potential input variables, such as the close relationship between each pollutant and the meteorological variables. Furthermore, the different combinations of the meteorological variables were tested, and we selected the combination with the best performance to predict the air pollutant concentrations based on the trained neural network and the corresponded predictors. Lagged air pollutant concentrations were included as a predictor variable. It is noted that the wind direction is replaced by the following, which has been calculated through:

(15) WD=1+sin(φ−π/4)

where *φ* is the wind direction in radians.

The experiments are carried out in MATLAB 2014 environment running in a Pentium 4, 1.9 GHZ CPU. We adopted 10-fold cross-validation to assess whether ELM can be generalized to an independent data set. Using the 10-fold cross validation (CV) scheme, the dataset was randomly divided into ten equal subsets. At each run, nine subsets were used to construct the model, while the remaining subset was used for prediction. The average results and the correlation coefficients are shown in Table 2. The average accuracy for 10 iterations was recorded as the final prediction. We use the training subset to learn and adjust the weights and biases of the predefined ELMs, and the testing subset is used to evaluate the generalization ability of the trained network. Generally, the larger the training set, more accurate models will be obtained.

In order to avoid the performance being dominated by any variables, we scaled the data set to commensurate data ranges, data including the inputs and the targets have been normalized into [−1,1]. The results of the models were reverse-scaled to compare the performance of MLR, FFANN-BP, and ELM. For FFANN-BP, we adopted the Levenberg–Marquardt algorithm, which is generally the fastest method for training moderate-sized FFANN. For ELM, the sigmoid function for the hidden layer and linear function for the output layer are used in our paper.

The number and selection of input variables are very important in the performance of the prediction of air pollutant concentration algorithms. For FFANN-BP and ELM, the number of hidden nodes are gradually increased. We selected the optimal number of nodes for FFANN-BP and ELM by cross-validation. The number of the hidden nodes for ELM and FFANN-BP was set as 20.

In order to evaluate the performance of the three methods, four statistical parameters were calculated, including mean absolute error (MAE), root mean square error (RMSE), the index of agreement (IA), and the coefficient of determination ($R^{2}$). RMSE, MAE, IA, and $R^{2}$ of the three models are shown in Table 2. The results with the highest $R^{2}$ value and the lower value of RMSE is the best method. RMSE is calculated as follows :

(16) $$MAE=\frac{\sum_{i=1}^{n}\left|O_{i}-T_{i}\right|}{n}$$

(17) $$RMSE=\sqrt{\frac{\sum_{i=1}^{n}{\left(O_{i}-T_{i}\right)}^{2}}{n}}$$

(18) $$R^{2}=\frac{\sum_{i=1}^{n}{\left(T_{i}-\bar{O}\right)}^{2}}{\sum_{i=1}^{n}{\left(O_{i}-\bar{O}\right)}^{2}}$$

(19) $$IA=1-\frac{\sum_{i=1}^{n}{\left(T_{i}-O_{i}\right)}^{2}}{\sum_{i=1}^{n}\left(\right|O_{i}-\bar{O}\left|+\right|T_{i}-\bar{O}{\left|\right)}^{2}}$$

where $O_{i}$ is the *i*-th corresponding observed concentration, $T_{i}$ is the *i*-th predicted concentration, $\bar{O}$ is the average of observation, and *n* is the number of data. Table 3 summarizes the performance of the derived models in the four sites in terms of the squared correlation coefficient ($R^{2}$) among the observed the observed and predicted values, the mean average error (MAE), the root mean square error (RMSE), and the index of agreement (IA).

### 4.1. Results

Firstly, the architectures for the four seasons of summer, monsoon, post-monsoon, and winter have been trained through MLR, FFANN-BP, and ELM based on daily data of 2010–2015. Hence, the forecasted values of daily air pollutant concentrations for the validated data have been compared with the observed values of the same time, as shown in Table 2. The $R^{2}$, RMSE, IA, and MAE were found to be better in summer than in all three seasons. The coefficients of determination ($R^{2}$) have almost significant values (0.70) in all seasons. The statistical analysis of the three models’ validation in the validated data have been shown in the same table, which reveals that ELM is performing satisfactorily with respect to RMSE and $R^{2}$ in summer, winter, post-monsoon, and monsoon, in decreasing order. However, we found that the ELM model obtained the best performance in terms of four statistical parameters. We found that root mean square error (RMSE) and mean absolute error (MAE) were better in summer than in all three seasons, the $R^{2}$ and IA were observed to be almost the same in all seasons.

#### 4.1.1. Coefficient of Determination

Based on the performance measures, ranking of the statistical models used in the present study have been done in Table 2. We selected the Sham Shui Po monitoring station to demonstrate the performance of the three methods. The coefficient of determination for $NO_{2}$ varied from 0.52 to 0.61 for MLR, 0.57 to 0.67 for FFANN-BP, and 0.65 to 0.71 for ELM for four seasons. The coefficient of determination for $NO_{x}$ varied from 0.54 to 0.66 for MLR, 0.56 to 0.76 for FFANN-BP, and 0.62 to 0.83 for ELM for four seasons. The coefficient of determination for $O_{3}$ varied from 0.54 to 0.59 for MLR, 0.59 to 0.60 for FFANN-BP, and 0.55 to 0.72 for ELM. The coefficient of determination for $PM_{2.5}$ varied from 0.50 to 0.64 for MLR, 0.52 to 0.67 for FFANN-BP, and 0.70 to 0.82 for ELM. The coefficient of determination for $SO_{2}$ varied from 0.55 to 0.74 for MLR, 0.54 to 0.71 for FFANN-BP, and 0.61 to 0.78 for ELM. The observations made in the study reveal that the ELM-based technique scored well over MLR and FFANN-BP. ELM is the most suitable statistical technique for the prediction of air pollutant concentrations. The results reveal that the performance of the statistical models is often superior to MLR and FFANN-BP for four seasons.

#### 4.1.2. RMSE

There appears to be very good agreement between the predicted and observed concentrations for three models. However, the ELM model yielded the lowest RMSE compared to the slightly higher values obtained by FFANN-BP and MLR. The ELM model performed best in terms of RMSE, which is in agreement with the coefficient of determination results. It is shown in Table 2 that the RMSE between the predicted and the observed concentrations for each air pollutant has the lowest values for ELM. However, for MLR and FFANN-BP, the RMSE is higher. A similar conclusion is drawn for mean absolute error. Clearly, ELM outperforms the other two counterparts in the testing phase. This indicates that the ELM model had a slightly better skill in the generalization. The same advantages of the three techniques is that they use a single type of data concentration and effort in training the data with meteorological, emission, and other such data—in comparison to other methods such as the numerical models. However, MLR can not capture the complex relationship of the data. This results in the poor performance of MLR. The high values of the index of agreement indicate a satisfying forecast of the daily average values of air pollutant concentration by the three models for four seasons.

#### 4.1.3. Speed

Moreover, it is shown in Table 3 that ELM performs better in terms of the learning speed against MLR and FFANN-BP. The greatest proportion of learning time of ELM is spent on calculating the Moore–Penrose generalized inverse of the hidden layer output matrix *H*. We run the efficient optimal FFANN-BP package provided by MATLAB2014 (MathWorks, Natick, MA, USA) for this application. The learning speed of ELM is faster than classic learning algorithms, which generally take a long time to train FFANN-BP. It is noted that ELM is the fastest compared to MLR and FFANN-BP. The experimental results show that ELM spent 0.183 s obtaining the testing RMSE 10.1; however, for FFANN-BP, it took 5 s to reach a much higher testing error of 15.8 for the $O_{3}$ concentration at Sham Shui Po. It can also be seen that ELM runs around 25 times faster than FFANN-BP, and eight times faster than MLR for the prediction of Hong Kong air pollutants. However, ELM only spent 0.05 s on learning, while FFANN-BP spent nearly 3 s on training. The underlying reason is that it is not necessary for ELM to iteratively search for the optimal solution. On the contrary, FFANN-BP obtains the optimal solution by gradient-based optimization.

#### 4.1.4. Generalization

**Figure 3:**
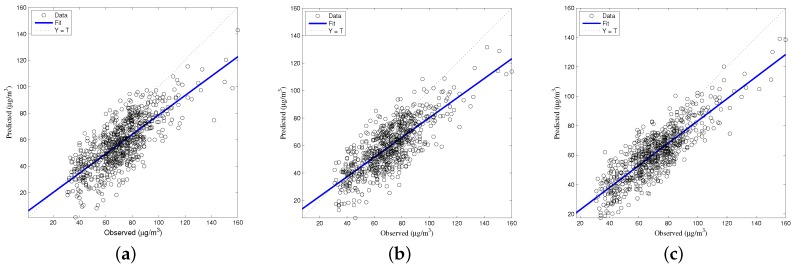
Comparison of prediction results among multiple linear regression (MLR), feedforward neural network based on back propagation (FFANN-BP), and extreme learning machine (ELM). NO_2_ predictions (**a**) MLR; (**b**) FFANN-BP; (**c**) ELM.

**Figure 4:**
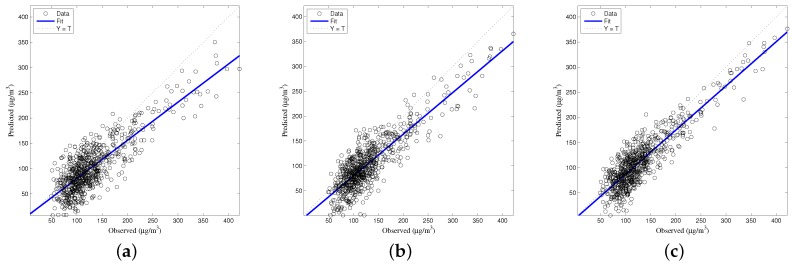
Comparison of prediction results among MLR, FFANN-BP, and ELM. NO_*x*_ predictions (**a**) MLR; (**b**) FFANN-BP; (**c**) ELM.

**Figure 5:**
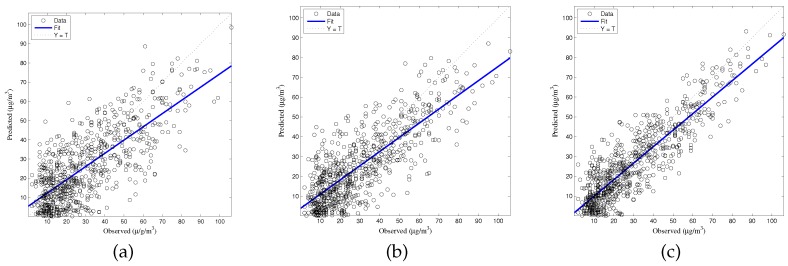
Comparison of prediction results among MLR, FFANN-BP, and ELM. O_3_ predictions (**a**) MLR; (**b**) FFANN-BP; (**c**) ELM.

**Figure 6:**
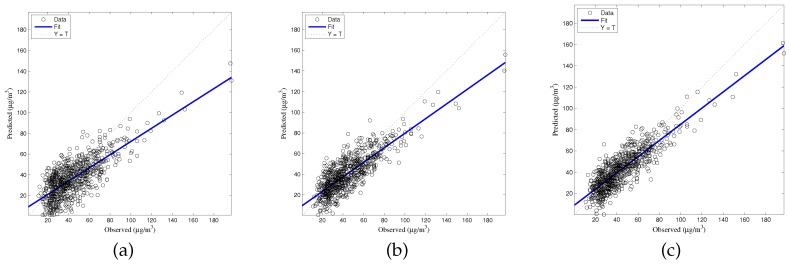
Comparison of prediction results among MLR, FFANN-BP, and ELM. PM_2.5_ predictions (**a**) MLR; (**b**) FFANN-BP; (**c**) ELM.

**Figure 7:**
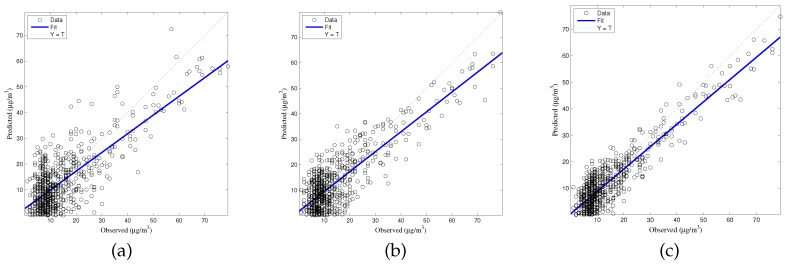
Comparison of prediction results among MLR, FFANN-BP, and ELM. SO_2_ predictions (**a**) MLR; (**b**) FFANN-BP; (**c**) ELM.

The generalized accuracy is estimated in our study. It is shown in Table 2 and Figure 3, Figure 4, Figure 5, Figure 6 and Figure 7 that the generalization of ELM is often better than gradient-based learning, as in FFANN-BP. FFANN-BP has some drawbacks, such as local minima and low convergence rate. It is shown that FFANN-BP falls into the trap of local minima. Some measures, such as weight decay and early stopping strategies are adopted to avoid these issues. In a reverse manner, ELM, reaching the solutions directly, is simpler than FFANN-BP. It is shown that the generalization ability of ELM is very stable with the number of the hidden nodes.

### 4.2. Episode

Different breakpoint concentrations and different air quality standards have been reported in the literature. In Hong Kong, to reflect the status of air quality and its effects on human health breakpoints have been considered for individual air pollutants; for example, for PM_2.5_ (0–50 μg/m^3^) “Low”, “High” (≥50 μg/m^3^). In summary, the “High” level is around 33.5%, and the percentage of “Low” level is around 66.5% in Hong Kong, respectively (about 3% of the data are NAs). The daily average values of days and the annual average value was persistently higher than the limit value of 50 μg/m^3^. Thus, the limit value of 50 μg/m^3^ was selected in order to verify the forecast quality of the developed models.

As was mentioned in the introduction, the concentration levels in Hong Kong center are considerable when compared to the standards imposed by the World Health Organization (WHO). The daily average values exceeded the limit value of 50 μg/m^3^ in 38% of days. Thus, the limit value of 50 μg/m^3^ was selected in order to verify the predicted quality of the ELM model. We selected the probability of detection (POD) and false alarm rate (FAR) indices in order to evaluate the prediction accuracy for the exceedances of the imposed limit. The POD and FAR should be reasonably high and low, respectively. It is shown in Table 4 that the three models fulfill these conditions to a large extent. Particularly, the ELM model can predict the exceedance and the non-exceedances accurately.

In order to show that the models can accurately predict the exceedances of the imposed limit, the values of POD and FAR should be reasonably high and low, respectively. The definitions of b, POD, PC and FAR are shown in the Formulas (20)–(23). As is exhibited in Table 4, these conditions are fulfilled by both models to a large extent. Moreover, the developed models can predict the exceedances and the non-exceedance to a satisfactory level.

(20) $$b=\frac{A+C}{A+B}$$

(21) $$POD=\frac{A}{A+B}$$

(22) $$PC=\frac{A+D}{A+B+C+D}$$

(23) $$FAR=\frac{C}{A+B}$$

where A, B, C, D represent the number of exceedances that were observed and forecasted, the number of exceedances that were observed but not forecasted, the number of exceedances that were not observed but forecasted and non-exceedances, respectively. Generally, the high levels of POD values show that the perfect performance of ELM in predicting the exceedances of PM_2.5_. Moreover, the FAR are found to be around 30%, the success rate of detection reach up to 91%. The lower performance of the RBF-NN shows that it is not appropriate for the prediction of the concentration of exceedances. Multilayer perceptron (MLP-NN) maps sets of input data onto a set of appropriate output. It provides powerful models which can distinguish data that are either nonlinearly. Radial basis function (RBF-NN) which is a neural network has radially symmetric functions in the hidden layer nodes. For RBF, the distance between the input vector and a prototype vector play an important role on the activation of the hidden neurons.

### 4.3. Comparison with Previous Studies

As stated above, during the last decade, many researchers used ANNs to forecast the particulate matter concentration levels in the ambient air pollution for Hong Kong, and numerous papers have been published. Some of them have focused on the prediction of hourly PM_2.5_ concentrations in Central and Mong Kong, Hong Kong [32,33], and proved the effectiveness of the proposed model. Specifically, Fei et al. [34] used to forecast hourly air pollutant NO_2_ concentrations in Hong Kong, and reported a correlation coefficient between modeled and measured concentrations around 0.70; there was a reasonably good agreement between the predicted and observed NO_*x*_ and O_3_ values. Zhao et al. (2003) [35] proposed the use of quantile and multiple line regression models for the forecasting of O_3_ concentrations in Hong Kong, and reported better performance, depending on the site, the training algorithm, the input configuration, etc. The results proved that the MLR worked better at suburban and rural sites compared to urban sites, and worked better in winter than in summer. Gong [36] proposed the combination of preprocessing methods and ensemble algorithms to effectively forecast ozone threshold exceedances, aiming to determine the relative importance of the different variables for the prediction of O_3_ concentration.

We also compare the performance of ELM with other similar methods in Table 5 [37,38,39,40,41].

## 5. Conclusions

In this paper, we proposed the prediction of the concentration of air pollutants based on ELM, due to the drawbacks of FFANN-BP, such as low convergence and their tendency to get caught in the local minimum. Compared with FFANN-BP, ELM overcomes the above drawbacks. ELM has several interesting and significant advantages compared with FFANN-BP which are based on a gradient learning algorithm.

It was shown that ELM performs well in terms of precision, robustness, and generalization. There are no significant differences between the prediction accuracies of each model. ELM provided the best performance on indicators related to goodness of the prediction, such as $R^{2}$ and RMSE, etc. The present study revealed that ELM perform slightly better than those of the simple statistical techniques.

## Figures and Tables

**Table 1:** The statistical description of four seasons in Sham Shui Po and Tap Mun, Hong Kong.

Air Pollutants	Season	Mean	Variance	Maximum	Minimum
NO_2_ (μg/m^3^)	Summer	70.7	20.0	182	31
	Monsoon	52.8	19.4	159	26
	Post-Monsoon	67.9	16.0	137	17
	Winter	75.7	21.4	185	27
NO_*x*_ (μg/m^3^)	Summer	129.1	57.7	513	49
	Monsoon	102.4	36.7	279	37
	Post-Monsoon	102.9	26.5	234	27
	Winter	132.7	61.1	601	31
O_3_ (μg/m^3^)	Summer	30.9	20.6	108	2
	Monsoon	21.4	15.7	122	2
	Post-Monsoon	44.6	23.8	118	4
	Winter	29.2	16.4	93	2
PM_2.5_ (μg/m^3^)	Summer	45.1	29.3	569	11
	Monsoon	28.8	14.8	116	11
	Post-Monsoon	49.6	20.7	143	9
	Winter	55.1	25.2	196	9
SO_2_ (μg/m^3^)	Summer	14.2	12.7	80	1
	Monsoon	15.2	12.5	84	1
	Post-Monsoon	11.5	8.2	62	0
	Winter	13.7	10.5	125	0
Daily Average Temperature (°C)	Summer	23.0	4.0	14.3	30.0
	Monsoon	29.3	1.0	25.2	31.2
	Post-Monsoon	26.4	2.8	19.8	30.5
	Winter	16.4	3.0	7.7	20.8
Relative Humidity (%)	Summer	85	7.9	67	99
	Monsoon	80.4	6.0	58	96
	Post-Monsoon	75.4	7.9	54	94
	Winter	71.9	13.4	29	95
Daily Max Temperature (°C)	Summer	25.8	4.4	15.4	32.8
	Monsoon	32.4	1.3	28.3	34.8
	Post-Monsoon	30.0	3.0	22.1	35.1
	Winter	20.3	3.4	9.2	27.1
Daily Min Temperature (°C)	Summer	20.8	3.9	13.2	28.5
	Monsoon	26.7	1.2	23.2	28.9
	Post-Monsoon	24.0	2.6	18.2	28.0
	Winter	13.6	3.0	6.3	18.8
Wind Speed (m/s)	Summer	24.4	9.9	7.0	53.6
	Monsoon	17.1	7.6	6.8	43.3
	Post-Monsoon	21.2	8.7	4.5	54.8
	Winter	26.9	9.0	3.9	52.2
Prevailing Wind Direction (°)	Summer	0.8	0.7	0.00	2.0
	Monsoon	1.1	0.7	0.03	2.0
	Post-Monsoon	0.7	0.7	0.03	2.0
	Winter	0.9	0.7	0.0	2.0

**Table 2:** The mean performance of multiple linear regression (MLR), feedforward neural network based on back propagation (FFANN-BP), and extreme learning machine (ELM) for Sham Shui Po and Tap Mun. RMSE: root mean square error; $R^{2}$: coefficient of determination; IA: index of agreement; MAE: mean absolute error.

Stations	Season	Air Pollutants	MLR				FFANN-BP				ELM			
			RMSE	*R*^2^	IA	MAE	RMSE	*R*^2^	IA	MAE	RMSE	*R*^2^	IA	MAE
Sham Shui Po	Summer	NO_2_	19.0	0.57	0.77	15.4	16.9	0.61	0.81	13.7	14.3	0.71	0.86	11.7
		NO_*x*_	41.0	0.69	0.85	33.1	37.8	0.75	0.88	30.7	30.8	0.80	0.92	24.9
		O_3_	14.5	0.56	0.85	11.4	13.2	0.64	0.88	10.4	10.1	0.78	0.93	8.0
		PM_2.5_	16.4	0.57	0.83	13.1	12.9	0.68	0.89	10.3	11.3	0.74	0.92	8.9
		SO_2_	7.9	0.62	0.88	6.2	6.9	0.71	0.91	5.4	5.4	0.84	0.95	4.3
	Monsoon	NO_2_	28.2	0.52	0.69	22.7	24.8	0.56	0.76	20.2	19.5	0.64	0.83	16.3
		NO_*x*_	44.3	0.62	0.76	31.6	36.3	0.66	0.82	29.2	28.3	0.74	0.92	21.4
		O_3_	30.3	0.54	0.70	24.2	20.3	0.56	0.78	16.2	17.3	0.60	0.85	13.7
		PM_2.5_	18.9	0.64	0.70	17.7	14.8	0.67	0.82	11.9	6.9	0.86	0.94	5.5
		SO_2_	18.1	0.54	0.69	19.6	16.9	0.60	0.74	13.6	10.6	0.67	0.86	8.5
	Post-Monsoon	NO_2_	28.1	0.61	0.69	24.7	23.8	0.67	0.76	19.2	17.2	0.69	0.86	13.9
		NO_*x*_	45.3	0.54	0.68	40.9	43.3	0.56	0.79	36.2	31.7	0.62	0.86	28.4
		O_3_	29.2	0.59	0.66	23.6	20.3	0.56	0.77	16.2	17.2	0.55	0.84	14.3
		PM_2.5_	23.4	0.50	0.69	27.7	19.8	0.52	0.74	21.9	17.7	0.72	0.83	13.2
		SO_2_	15.1	0.55	0.62	14.6	11.9	0.54	0.71	11.6	8.1	0.61	0.77	7.0
	Winter	NO_2_	31.2	0.60	0.61	27.7	28.5	0.66	0.68	25.2	21.2	0.71	0.74	18.9
		NO_*x*_	43.3	0.56	0.72	40.1	40.7	0.63	0.79	36.2	39.0	0.77	0.91	27.6
		O_3_	26.3	0.58	0.69	24.2	18.3	0.60	0.76	16.2	19.3	0.72	0.83	15.7
		PM_2.5_	25.4	0.60	0.69	20.7	21.9	0.67	0.76	18.8	18.2	0.71	0.89	15.0
		SO_2_	13.7	0.74	0.77	14.6	15.8	0.71	0.79	10.6	7.1	0.62	0.87	5.8
Tap Mun	Summer	NO_*x*_	25.6	0.64	0.69	22.7	20.6	0.70	0.74	18.4	19.2	0.73	0.79	16.7
		NO_*x*_	35.5	0.65	0.80	30.3	27.2	0.71	0.86	26.6	25.7	0.72	0.91	23.7
		O_3_	23.4	0.64	0.76	18.5	15.7	0.79	0.85	11.2	12.3	0.84	0.90	10.9
		PM_2.5_	26.2	0.69	0.76	22.1	19.8	0.74	0.81	17.6	17.9	0.79	0.84	15.3
		SO_2_	13.1	0.69	0.76	10.2	9.9	0.74	0.86	7.6	7.3	0.85	0.91	5.9
	Monsoon	NO_2_	25.9	0.67	0.72	22.7	25.2	0.66	0.71	23.2	20.1	0.75	0.78	18.2
		NO_*x*_	36.8	0.61	0.68	31.6	30.4	0.69	0.73	27.3	27.7	0.74	0.79	24.9
		O_3_	25.7	0.62	0.68	19.2	20.1	0.71	0.75	18.5	17.6	0.79	0.82	14.8
		PM_2.5_	17.4	0.65	0.70	12.7	14.8	0.69	0.77	11.9	10.1	0.76	0.81	8.5
		SO_2_	14.9	0.65	0.79	13.4	13.6	0.79	0.83	11.2	7.5	0.84	0.89	6.9
	Post-Monsoon	NO_2_	27.7	0.65	0.70	24.2	23.2	0.76	0.82	18.3	17.6	0.80	0.87	15.7
		NO_*x*_	38.8	0.69	0.73	34.3	35.2	0.74	0.79	30.2	30.4	0.81	0.86	28.6
		O_3_	26.3	0.54	0.58	24.2	18.3	0.59	0.63	16.4	17.6	0.74	0.77	11.8
		PM_2.5_	28.3	0.70	0.75	26.2	23.9	0.77	0.81	21.9	17.6	0.82	0.89	15.8
		SO_2_	13.2	0.74	0.80	11.1	10.0	0.76	0.88	9.2	7.2	0.89	0.91	6.4
	Winter	NO_2_	35.1	0.58	0.63	32.6	30.8	0.67	0.69	27.7	26.4	0.72	0.77	30.6
		NO_*x*_	38.9	0.55	0.60	31.7	32.4	0.61	0.63	28.2	26.7	0.67	0.72	24.9
		O_3_	31.8	0.62	0.72	29.6	28.4	0.74	0.79	25.6	25.7	0.77	0.82	20.7
		PM_2.5_	29.6	0.64	0.75	25.3	25.8	0.77	0.81	20.1	20.1	0.82	0.86	16.5
		SO_2_	12.4	0.69	0.75	10.6	11.7	0.72	0.80	9.3	7.7	0.79	0.83	6.1

**Table 3:** The training time *s* of MLR, FFANN-BP, and ELM on the air pollutant O_3_ with the size of the hidden layers 20 at Sham Shui Po.

Air Pollutants	MLR	FFANN-BP	ELM
NO_2_	0.25	5.11	0.05
NO_*x*_	0.27	4.96	0.06
O_3_	0.33	7.38	0.07
SO_2_	0.26	6.41	0.05
PM_2.5_	0.44	6.38	0.06

**Table 4:** The mean predicting performance of the exceedance for the air pollutant PM_2.5_ for RBF-NN, MLP-NN, and ELM. b: bias; POD: probability of detection; PC: the percentage correct; FAR: false alarm rate.

Statistical Measure	RBF-NN	MLP-NN	ELM
b	0.39	0.86	0.95
FAR	0.24	0.31	0.27
POD	0.22	0.67	0.73
PC	0.86	0.87	0.91

**Table 5:** The mean performance of other similar methods. RMSE: root mean square error; $R^{2}$: coefficient of determination.

Publication	Area	Air Pollutant	$R^{2}$	RMSE	Methodology
Bougoudis et al. (2016) [37]	Athens	SO_2_	0.75	8.30	Combined machine learning algorithm
Paschalidou et al. (2011) [38]	Limassol, Cyprus	PM_10_	0.33	26.2	PCA-RBF
Papaleonidas and Iliadis. (2013) [39]	Athens	O_3_	0.71	15.2	Neurocomputing
Kumar and Goyal. (2013) [40]	Delhi	Air Quality Index	0.77	32.1	PCA-NN
Azid et al. (2014) [41]	Malaysia	Air Quality Index	0.615	10.0	FFANN-BP PCA
